# Supplementary material for: Innovative Alignment-Based Method for Antiviral Peptide Prediction
Source: Antibiotics (Basel). 2024 Aug 14;13(8):768. doi: 10.3390/antibiotics13080768 (PMC11350826; doi:10.3390/antibiotics13080768)
Supplement: Supplementary file 1 [file antibiotics-13-00768-s001.zip › SuppInfo/SI1_Supporting_Tables .pdf]

Innovative Alignment-Based Method for Antiviral Peptide Prediction

Daniela de Llano García[1], Yovani Marrero-Ponce[2,3*], Guillermin Agüero-Chapin[4,5*], Agostinho Antunes[4,5], Felix Martinez-Rios[3] and Hortensia Rodriguez[1]

1 School of Chemical Sciences and Engineering, Yachay Tech University, Hda. San José s/n y Proyecto Yachay, Urcuquí 100119, Ecuador
2 Universidad San Francisco de Quito (USFQ), Grupo de Medicina Molecular y Traslacional (MeM&T), Colegio de Ciencias de la Salud (COCSA), Escuela de Medicina, Edificio de Especialidades Médicas; and Instituto de Simulación Computacional (ISC-USFQ), Diego de Robles y vía Interoceánica, Quito, 170157, Pichincha, Ecuador
3 Universidad Panamericana, Facultad de Ingeniería, Ciudad de México, México
4 CIIMAR – Centro Interdisciplinar de Investigação Marinha e Ambiental, Universidade do Porto, Terminal de Cruzeiros do Porto de Leixões, Av. General Norton de Matos, s/n, 4450-208, Portugal
5 Departamento de Biologia, Faculdade de Ciências, Universidade do Porto, Rua do Campo Alegre, 4169-007 Porto, Portugal

* Correspondence: ymarrero@usfq.edu.ec (Y.M.-P.); gchapin@ciimar.up.pt (G.A.-C.)

Contents

Table SI1.1 Brief Description of the initial scaffolds used for the MQSSMs 2

TableSI1.2 Parameters used for selected MQSSMs 3

Tab SI1.3 Final Overall Ranking 4

Tab SI1.4 Web server and Stand-Alone Software for the prediction of AVPs 5

Table SI1.1 Brief Description of the initial scaffolds used for the MQSSMs

Name	Size	Description	Name	Size	Description
Md1	1872	Merged of scaffolds with 50% from scaffold extraction	SG4	2562	Constructed using Global Alignment, HB Similarity Measure and 80% sequence identity
Md2	2152	Merged of scaffolds with 60% from scaffold extraction	SG5	3119	Constructed using Global Alignment, HB Similarity Measure and 90% sequence identity
Md3	2445	Merged of scaffolds with 70% from scaffold extraction	SL1	1030	Constructed using Local Alignment, HC Similarity Measure and 50% sequence identity
Md4	2703	Merged of scaffolds with 80% from scaffold extraction	SL2	1557	Constructed using Local Alignment, HC Similarity Measure and 60% sequence identity
Md5	3206	Merged of scaffolds with 90% from scaffold extraction	SL3	2028	Constructed using Local Alignment, HC Similarity Measure and 70% sequence identity
SG1	1626	Constructed using Global Alignment, HB Similarity Measure and 50% sequence identity	SL4	2369	Constructed using Local Alignment, HC Similarity Measure and 80% sequence identity
SG2	1991	Constructed using Global Alignment, HB Similarity Measure and 60% sequence identity	SL5	3003	Constructed using Local Alignment, HC Similarity Measure and 90% sequence identity
SG3	2277	Constructed using Global Alignment, HB Similarity Measure and 70% sequence identity			

TableSI1.2 Parameters used for selected MQSSMs

Model	Alignment	Identity %	Scaffold
Base Models			
M1	global	50	Md3
M2	global	50	Md4
M3	global	70	Md4
M4	local	70	SG4
M5	local	80	SL5
M6	global	40	SG4
M7	local	90	SL5
M8	global	40	SG5
M9	global	50	SG5
M10	local	70	SG5
M11	local	80	SG5
M12	local	90	SG5
Modified Models			
M13	global	90	Fusion
M3+	global	70	Md4+
M7+	local	90	SL55+
M12+	local	90	SG5+
M13+	global	90	Fusion+
E1	global	90	EG5
E2	global	90	EL5

Table SI1.3 Final Overall Ranking

Predictor	Friedman Ranking	Predictor	Friedman Ranking
M13+	4.6	Meta	11.6
EI	5	PTPAMP	11.6
M7	5.6	AntiVPP	11.6
M12	5.6	ProtDcalHier	11.8
M3+	6.4	AMPfun	12.2
LACVP	8	PC6	12.6
AVPpred	9	ClassAMP	13.2
FIRM	11	ProtDcalRNN	13.4
ProtDcalRF	11.2	seqpros	14.2

Table SI1.4 Web server and Stand-Alone Software for the prediction of AVPs

Name	Year	Algorithm	Encoding	Implementation	Ref
iACVP	2022	RF	word-embedding word2vec	http://kurata35.bio.kyutech.ac.jp/iACVP/	(1)
AI4AVP	2022	CNN	PC6, ENNAVIA descriptor , AAC, PseACC, AA index, DPC	https://axp.iis.sinica.edu.tw/AI4AVP/	(2)
PTPAMP	2022	SVM	AAC, DPC	http://www.nipgr.ac.in/PTPAMP/	(3)
Deep-AVPpred	2022	ANN	Pretained Embeddings	https:// deep- avppr ed. anvil. app/	(4)
AVPIden	2021	Sharpley Value	Phyc, PAAC, DPC, AAC, CKSAAGP	https://awi.cuhk.edu.cn/AVPIden/#/	(5)
ENNAVIA	2021	NN	AAC, DPC, TPC, Reduced AAC, GDC, GTC, CTD, PseACC, Phyc, AA index , steric hindrance , bulkiness, secondary strcuture propensities, side chain interactions, membrane buried preference parameters	https://research.timmons.eu/ennavia	(6)
ProtDcal	2021	RF, RNN	GTPC,APseAAC, PseACC, GAAC,ACC, PHYS	https://biocom-ampdiscover.cicese.mx/	(7)
AMPfun	2020	RF	AAC-bas,Phyc, and word frequency-based features	http://fdblab.csie.ncu.edu.tw/AMPfun/index.html	(8)
Meta-iAVP	2019	RF	AAC, Am-PseAAC	http://codes.bio/meta-iavp/	(9)
PEPred-Suite	2019	RF	AAC, ASDC,CTD,188D, GGAP,BIT20, BIT21,OLP,IT,DC	http://server.malab.cn/PEPred-Suite.	(10)
MLAMP	2016	RF, ML-SMOTE	PseAAC	http://www.jci-bioinfo.cn/MLAMP	(11)
AVP-IC50Pred	2015	SVM, RF, IBk, kStar	IC50	http:// crdd.osdd.net/servers/ic50avp/	(12)
iAMP-2L	2013	FKNN	PseAAC	http://www.jci-bioinfo.cn/iAMP-2L.	(13)
ClassAMP*	2012	RF, SVM	AAC,Phyc, BLOSUM 50 MATRIX, normalized van der Waals volume, secondary structure propensity, DPC, TPC, CTD	http://www.bicnirrh.res.in/classamp/	(14)
AVPpred	2012	SVM	PseAAC	http://crdd.osdd.net/servers/avppred/	(15)

LSTM_pep	2023	LSTM	-	https://github.com/haiping1010/New_peptide_iteration	
Pep-CNN	2022	CNN	AAC,Phys,evolutionary derived	https://github.com/alivelxj/Pep-CNN	(16)
seqpros	2022	MLP,LSTM	Phys	https://github.com/eotovic/seqprops_therapeutic.	(17)
TransImbAMP	2022	Transformer	-	https://github.com/ BiOmicsLab/TransImbAMP	(18)
PreAntiCoV	2021	RF	AAC,DPC,K-spaced AA,PseAAC,Phys	https://github.com/poncey/PreAntiCoV	(5)
iAMP-CA2L	2021	CNN-BiLSTM-SVM	cellular automata image	https://github.com/liujin66/iAMP-CA2L/tree/main	(19)
FIRM-AVP	2020	RF, SVM, DL	Aac, DC, APseAAC,CTD, secondary structure	https://github.com/pmartR/FIRM-AVP	(20)
AntiVPP	2019	RF	Phys, AAC	https://github.com/bio-coding/AntiVPP	(21)
